# Comparison of platelet‐rich fibrin with zinc oxide eugenol in the relief of pain in alveolar osteitis

**DOI:** 10.1002/hsr2.354

**Published:** 2021-08-11

**Authors:** Satheesh Reeshma, Chacko Pearl Dain

**Affiliations:** ^1^ Department of Oral and Maxillofacial Surgery Government Dental College Trivandrum India

**Keywords:** alveolar osteitis, pain relief, platelet rich fibrin, VAS, zinc oxide eugenol

## Abstract

**Background and aims:**

Alveolar osteitis (AO) is the most common painful post‐operative complication after tooth extraction. The common modalities used in the management of AO are lavage, placement of medicated dressings, analgesics, and antibiotics. The present study was undertaken to compare platelet‐rich fibrin (PRF) and zinc oxide eugenol (ZOE) for pain relief in AO.

**Methods:**

All cases meeting the eligibility criteria received two different treatment modalities over a span of 18 months. At the analysis stage, the final sample size comprised 70 patients, with 35 patients appropriated in each group. Group A patients received ZOE and Group B received PRF. Pain scores were measured on “1st, 3rd, 5th, and 7th” days based on a visual analogue scale (VAS) and compared in both groups of patients. The collected data were analyzed using the chi‐square test, *t* test, and Mann‐Whitney *U* test.

**Results:**

In patients treated with ZOE dressing, the average VAS scores observed were 7.4 ± 1.5, 5.1 ± 1.1, 3.4 ± 0.9, and 2.1 ± 0.7, respectively, on the “1st, 3rd, 5th, and 7th” follow‐up days. In patients treated with PRF, the average VAS score observed were 4.1 ± 1.2, 2.6 ± 0.9, 1.7 ± 0.9, and 0.8 ± 0.8 respectively.

**Conclusion:**

Both ZOE and PRF were effective in pain control during the follow‐up period. However, the pain intensity measured as a pain score using VAS was, lower in the PRF group than in the ZOE group on all follow‐up days.

## 1 INTRODUCTION

Alveolar osteitis (AO) commonly known as “dry socket” is a painful condition of jaws that typically begins 1 to 3 days following extraction of teeth. Blum defined dry socket as “postoperative pain in and around the extraction site, which increases in severity at any time between 1 and 3 days after the extraction, accompanied by a partially or totally disintegrated blood clot within the alveolar socket, with or without halitosis” while, excluding other possible causes of pain in the region.^1^ This condition may persist for 5 to 10 days and resolve on its own. The clinical features of AO are intense throbbing pain in and around the extraction socket, often radiating in nature, extraction socket devoid of clot which may appear totally empty or partly covered with a grayish‐yellow membrane of necrotic tissue, fetid breath, bad taste, gingival edema, and localized lymphadenitis.^2^, ^3^

A multifactorial etiology has been suggested for the dry socket which includes oral anaerobic microorganisms, anatomic location, traumatic extractions, immoderate use of irrigating agents at the surgical site, curettage of the extraction socket, mechanical dislodgement of blood clot, fibrinolysis of blood clot, smoking, oral contraceptives, immune suppression, female gender, and vasoconstrictors.^2^, ^4^, ^5^, ^6^, ^7^, ^8^, ^9^

The emergence of AO as a common postoperative sequela of extraction has prompted surgeons to explore various treatment options, focusing on optimal healing and control over pain. It is fair to argue that the primary aim in the management of AO should be pain control until normal reparative processes are initiated.^10^ The common modalities used in the management of AO are alveolar lavage, placement of medicated dressings, analgesics, antibiotics topical anesthetics, and obtundent, or their combinations.^1^, ^2^, ^5^, ^9^ Nevertheless, the application of dressing materials inside the extraction socket has been reported to delay wound healing and cause adverse reactions.^3^, ^11^, ^12^ A plethora of pharmaceutical agents such as zinc oxide eugenol (ZOE), iodoform, chlorhexidine, butylparaminobenzoate, acemannan, guaiacol, chlorobutanol, neocone, and alvogyl are available for use in dry sockets. The sedative, antibacterial, and obtundent properties of ZOE have been utilized in the management of dry sockets, as intra‐alveolar dressings.^13^, ^14^

Platelet‐rich fibrin (PRF) is a second‐generation platelet concentrate developed by Choukroun et al in 2001.^15^ PRF is a tetra molecular polymer gel that incorporates platelets, leukocytes, cytokines, growth factors, and circulating stem cells into its matrix, which can accelerate physiologic wound healing and the formation of new bone.^16^, ^17^, ^18^, ^19^, ^20^, ^21^, ^22^, ^23^, ^24^, ^25^ The use of PRF has been postulated to accelerate the healing process of the extraction socket and in turn reduce postoperative pain.^26^, ^27^

The definitive management of dry sockets remains inconclusive. It has been felt that a meaningful study must be carried out to evaluate the relative effectiveness of two different modalities used for pain management in AO. The present study aimed to compare ZOE and PRF for pain relief in AO.

## 2 PATIENT AND METHODS

1. Study design

The design of the study was single‐blinded prospective study.

2. Sample selection procedure

**FIGURE 1:**
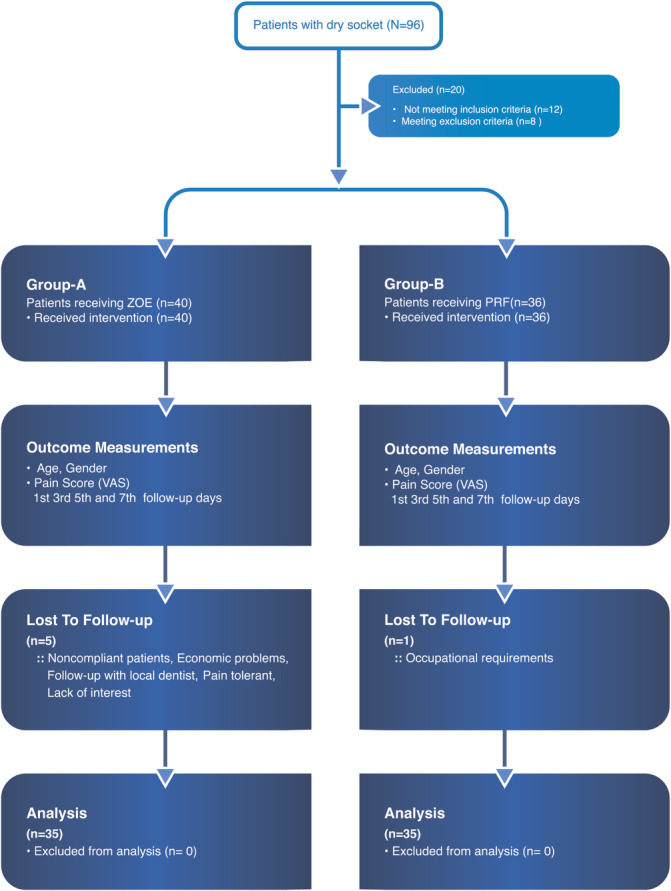
Flow chart showing the phases of prospective comparative study

All cases meeting the eligibility criteria received two different treatment modalities over 18 months. The methods of screening, grouping, interventions, follow‐up, and analysis are summarized in the flow diagram (Figure 1). Consequently, at the analysis stage, the final sample size comprised 70 patients, with 35 patients appropriated in each group. Group A patients received ZOE as the interventional modality and Group B received PRF.

3. Inclusion criteria

Patients diagnosed with AO following extraction of mandibular first and second molars without any active infection.

4. Exclusion criteria

1. Patients with known systemic illness, immunocompromised patients, smokers, patients with poor glycemic control, patients on steroid therapy, patients taking oral contraceptives, and pregnant and lactating women.
2. Patients undergoing third molar extractions.

5. Estimation of sample size^16^

$$N=\frac{2{\left(Z_{\alpha }+Z_{\beta }\right)}^{2}\sigma^{2}}{\Delta^{2}}$$

where *Z*_*∞*_ = 1.96 for ∞ = 0.05, *Z*_*β*_ = 0.84 for *β* = 0.20, Δ = *μ*_T_ − *μ*_C_ (difference in mean). *σ* = SD.

In this study,

Pooled SD of VAS score at seventh day (*σ*) = 0.495.

Mean différence in VAS score on seventh day between methods (Δ) = 0.33

$$N=\frac{2{\left(1.96+0.84\right)}^{2}\times {\left(0.495\right)}^{2}}{{\left(0.33\right)}^{2}}=35$$

Minimum sample size is 35 in each group. The final sample size is 70.

6. Outcome measures

1. Age
2. Gender
3. Pain based on visual analog scale (VAS)

### 2.1 Procedure

The following features were clinically diagnostic of AO.

1. Intense throbbing pain in relation to the extraction socket often radiates in nature and tends to increase in severity for a period between 1‐ and 3‐days post‐surgery.
2. Extraction socket denuded the blood clot with or without halitosis.

Group A patients received ZOE and Group B received PRF as the treatment modality. Pain score was measured on “1st, 3rd, 5th, and 7th” days based on a VAS and compared in both groups.

In group A patients, ZOE was used as an intra‐alveolar obtundent dressing (Vishal Dentocare Private Limited, India). (Contents: powder‐zinc oxide—80%, polymethyl methacrylate—20%, zinc stearate‐traces, and zinc acetate‐traces. Liquid‐eugenol—85%, and olive oil—15%).

Copious irrigation of the extraction socket was undertaken using a mixture of isotonic saline and povidone‐iodine solution, to clear the socket of any remnants of disintegrated blood clots and debris. A thin paste of ZOE was soaked in a gauze piece and placed in the extraction socket, adhering to aseptic measures. The dressings were changed on “1st, 3rd, 5th, and 7th” days.

#### 2.1.1 PRF protocol

**FIGURE 2:**
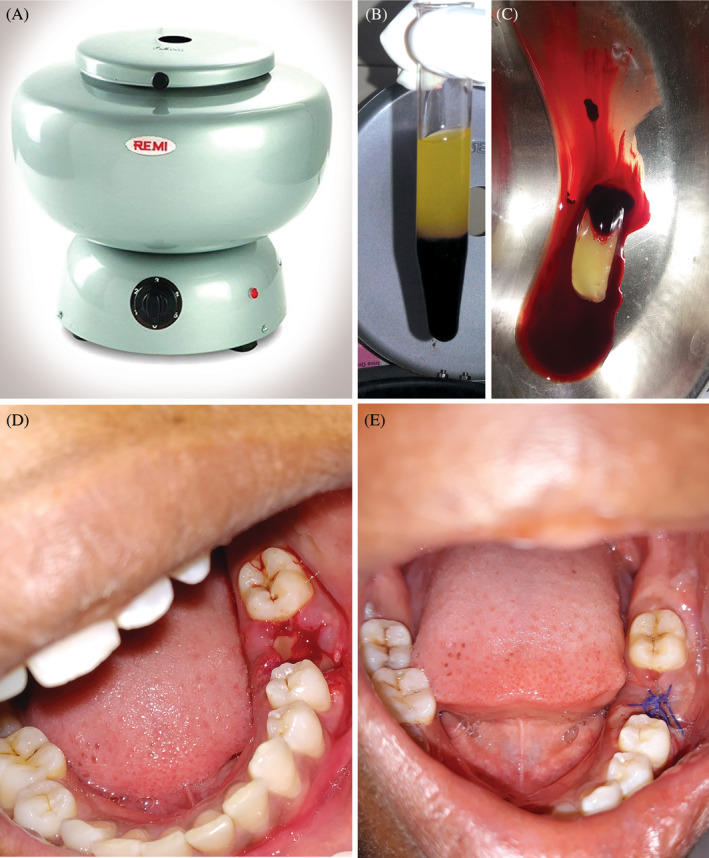
(A) Centrifuge used for platelet‐rich fibrin extraction (Remi clinical centrifuge C‐854/4, Remi Elektrotechnik Limited, India). (B) Blood after centrifugation. (C) Platelet‐rich fibrin. (D) Platelet‐rich fibrin placed inside the socket. (E) Platelet‐rich fibrin‐treated wound on 7th postoperative day

Samples were collected in an autoclavable test tube without anticoagulant and immediately centrifuged at 3500 rpm for 15 minutes until a thick, viscous, fibrous gel was obtained (Remi clinical centrifuge C‐854/4, Remi Elektrotechnik Limited, India) (Figure 2A). Centrifugation leads to the activation of platelets, which in turn release various coagulation cascades, resulting in fibrin formation. After centrifugation, the blood cells were sedimented at the bottom and plasma surfaces at the top. The supernatant plasma, which is poor in growth factors, is disposed of. The fibrin clot, which is rich in growth factors, is obtained in the middle of the tube between blood cells at the bottom and the surface plasma (Figure 2B, C). The platelet concentration in PRF is 4 to 8 times higher than the peripheral blood platelet concentration.^28^

In all group B patients, local anesthesia was administered using 2% lignocaine with adrenaline at a 1:200,000 concentration. Thorough irrigation of the extraction socket was performed using a mixture of isotonic saline and povidone‐iodine solution. PRF was then placed in the unhealed socket, and the surgical site was closed using a 3‐0 black silk suture. PRF‐treated wounds on seventh post‐operative day demonstrated satisfactory wound healing (Figure 2D, E).

### 2.2 Statistical analysis

The data were entered into Microsoft Office Excel 2007 version and the data were analyzed using SPSS for Windows, Version 16.0.* Qualitative variables are expressed as means, frequencies, and percentages. Qualitative variables were analyzed using the *t* test, chi‐square test, and Mann‐Whitney *U* test, and a *P* value <.05, which was defined as the level of statistical significance. SPSS Inc. Released 2007. SPSS for Windows, version 16.0. Chicago. SPSS Inc.

## 3 ETHICS

The study was approved by the Institutional Ethics Committee, Government Dental College, Thiruvananthapuram, Kerala, India (IEC/E/10/2017/DCT/ dated/November 27, 2017). Informed consent was obtained from all the participants.

## 4 RESULTS

The results are summarized in Tables 1, 2, 3. The maximum number of AO patients were in the age group of 30 to 39 years. The total number of male patients was 17 and female patients were 53. Table 1 summarizes the percentage distribution of AO among the different age groups and genders. In Group A patients treated with ZOE, 8 (22.9%) patients were below 30 years of age, 12 (34.3%) were in the range of 30 to 39 years of age; 7 (20.0%) range 40 to 49 years; 4 (11.4%) range 50 to 59; and 4 (11.4%) patients were, and ≥60 years, respectively. In Group B patients treated with PRF, 9 (25.7%) patients were below 30 years of age, 9 (25.7%) patients in the range of 30 to 39 years of age, 8 (22.9%) patients in the range of 40 to 49 years of age, 7 (20.0%) patients in the range of 50 to 59 years, and 2 (5.7%) patients were aged 60 years or above. The maximum number of patients treated with ZOE and PRF were in the age group of 30‐39. There was no significant difference in the mean age of the patients in both groups. Hence, the two study groups were homogenous in terms of age.

**TABLE 1:** Comparison of age and gender based on groups

		Zinc oxide eugenol		Platelet‐rich fibrin		*P*
		Count	Percent	Count	Percent	
Age	<30	8	22.9	9	25.7	.916
	30‐39	12	34.3	9	25.7	
	40‐49	7	20.0	8	22.9	
	50‐59	4	11.4	7	20.0	
	≥60	4	11.4	2	5.7	
	Mean ± SD	39.6 ± 14.2		39.3 ± 13		
Sex	Male	8	22.9	9	25.7	.780
	Female	27	77.1	26	74.3	

**TABLE 2:** Percentage distribution of the pain score between treatment groups

Pain score	Day 1				Day 3				Day 5				Day 7			
	ZOE		PRF		ZOE		PRF		ZOE		PRF		ZOE		PRF	
	Count	%	Count	%	Count	%	Count	%	Count	%	Count	%	Count	%	Count	%
0	0	0.0	0	0.0	0	0.0	0	0.0	0	0.0	0	0.0	0	0.0	17	48.6
1	0	0.0	0	0.0	0	0.0	1	2.9	0	0.0	16	45.7	7	20.0	9	25.7
2	0	0.0	3	8.6	0	0.0	19	54.3	4	11.4	14	40.0	16	45.7	9	25.7
3	0	0.0	7	20.0	1	2.9	9	25.7	19	54.3	3	8.6	12	34.3	0	0.0
4	0	0.0	15	42.9	11	31.4	4	11.4	9	25.7	2	5.7	0	0.0	0	0.0
5	7	20.0	4	11.4	12	34.3	2	5.7	1	2.9	0	0.0	0	0.0	0	0.0
6	3	8.6	6	17.1	9	25.7	0	0.0	2	5.7	0	0.0	0	0.0	0	0.0
7	6	17.1	0	0.0	1	2.9	0	0.0	0	0.0	0	0.0	0	0.0	0	0.0
8	9	25.7	0	0.0	0	0.0	0	0.0	0	0.0	0	0.0	0	0.0	0	0.0
9	9	25.7	0	0.0	1	2.9	0	0.0	0	0.0	0	0.0	0	0.0	0	0.0
10	1	2.9	0	0.0	0	0.0	0	0.0	0	0.0	0	0.0	0	0.0	0	0.0

**TABLE 3:** Comparison of pain between groups at different time interval

Pain	Zinc oxide eugenol		Platelet‐rich fibrin		*Z* ^a^	*P*
	Mean ± SD	Median	Mean ± SD	Median		
Day 1	7.4 ± 1.5	8	4.1 ± 1.2	4	6.5	<.01
Day 3	5.1 ± 1.1	5	2.6 ± 0.9	2	6.55	<.01
Day 5	3.4 ± 0.9	3	1.7 ± 0.9	2	5.94	<.01
Day 7	2.1 ± 0.7	2	0.8 ± 0.8	1	5.45	<.01

^a^ Mann‐Whitney *U* test.

In the present study, among 35 patients in group A, 8 (22.9%) were men and 27 (77.1%) were women. In group B, 9 (25.7%) patients were men and 26 (74.3%) were women. The incidence of AO was more common in females among the treatment groups. There was no significant difference in the number of male and female patients treated in either group. Hence the two study groups were homogenous in terms of sex.

**FIGURE 3:**
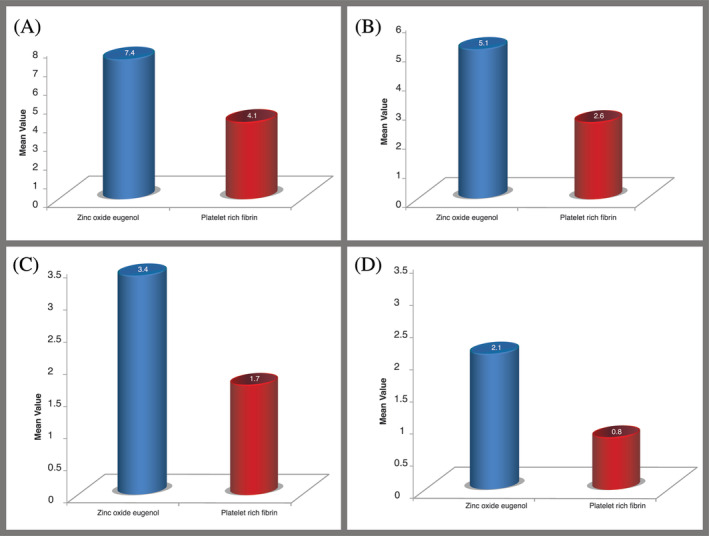
Bar diagram showing percentage distribution of the mean value of pain score between treatment groups on (A) Day 1, (B) Day 3, (C) Day 5, and (D) Day 7

Comparison of pain scores between the two groups on days 1, 3, 5, and 7 are summarized in Table 2. The mean and median pain scores at different time intervals are shown in Table 3. On day 1, comparing the two groups, group A had a mean pain score of 7.4 ± 1.5, and group B had a mean pain score of 4.1 ± 1. 2. The pain relief obtained in the PRF group on day 1 was statistically significant (*P* < .01) (Figure 3A).

On day 3, comparing the two groups, group A had a mean pain score of 5.1 ± 1.1, and group B had a mean pain score of 2.6 ± 0.9. The pain relief obtained in the PRF group on day 3 was statistically significant (*P* < .01) (Figure 3B).

On day 5, comparing the two groups, group A had a mean pain score of 3.4 ± 0.9, and group B had a mean pain score of 1.7 ± 0.9. The pain relief obtained in the PRF group was statistically significant (*P* < .01) on day 5 (Figure 3C).

On day 7, comparing the two groups, group A had a mean pain score of 2.1 ± 0.7 and group B had a mean pain score of 0.8 ± 0.8. The pain relief obtained in the PRF group was statistically significant (*P* < .01) on day 7 (Figure 3D).

## 5 DISCUSSION

AO is one of the most common and unpleasant postoperative complications following extraction. It was first described by Crawford in 1896. Birn's hypothesis^4^ is the most accepted explanation of dry socket to date expounding the significance of the localized fibrinolytic activity. According to this hypothesis, trauma and inflammation cause the release of tissue activators from adjacent tissues. Tissue activators convert plasminogen (present in the blood clot) to plasmin which causes lysis of blood clots and kininogen to kinin which causes pain.^4^, ^12^, ^14^, ^29^, ^30^

In the present study, the maximum number of patients was group**—**30 to 39 years, and the minimum number was reported in the sixth decade. This is in agreement with previous studies where the common age group was reported in the range of 30 to 40 years.^14^, ^16^ However, some studies have reported a peak incidence in the second decade.^31^, ^32^

Sweet and Butler suggested an increased incidence of AO with the use of oral contraceptives and found a positive correlation between them.^33^ Catellani et al observed that this increased incidence could be due to the higher concentration of estrogen contained in them.^34^ In the present study, 17 patients were men and 53 were women. Thus, the occurrence of AO showed a female preponderance, since patients taking oral contraceptives were excluded from this study, the predilection in female patients could not be associated with the use of oral contraceptives. This could be attributable to elevated endogenous estrogen levels during reproductive age.

The etiopathogenesis of AO remains inconclusive. Hence, the management of dry sockets lies in the paradigm between intra‐alveolar dressings and minimally invasive procedures.

The use of intra‐alveolar dressings such as salicept patch, neocon, alvogyl, and ZOE have demonstrated positive outcomes regarding pain relief in AO patients.^3^, ^9^, ^14^, ^35^ However, each of these dressings is characterized by distinct merits and demerits.^36^, ^37^ ZOE is a commonly used obtundent material with antibacterial properties. Adverse reactions to eugenol have been reported to vary from local reactions to anaphylaxis.^11^, ^37^ However, in the present study, 35 patients treated with ZOE failed to demonstrate any adverse reactions.

Various studies have shown that PRF is a natural fibrin‐based biomaterial that accelerates the healing mechanism of tissues and reduces inflammation. PRF is a fibrin matrix of trapped platelets, cytokines, and other cells that act as bio‐resorbable membranes. The activation and degranulation of these cells release various growth factors such as transforming growth factor‐beta, platelet‐derived growth factor, vascular endothelial growth factor, epidermal growth factor, fibroblast growth factor, and so on. These factors initiate the healing process by stimulating cell migration and proliferation within the fibrin matrix. They also play a role in angiogenesis, chemotaxis, granulation tissue production, epithelization, and osteogenesis.^19^, ^24^, ^38^, ^39^, ^40^, ^41^

Hussain et al concluded that PRF is as effective as ZOE in the management of AO for pain control and superior to ZOE in terms of socket healing and anti‐inflammatory properties.^16^ In a study comparing the efficacy of alvogyl and ZOE, Supe et al concluded that alvogyl required the least number of dressings and provided quicker and lasting pain relief and faster recovery to the patients.^9^ Faizel et al compared neocone, alvogyl, and ZOE and reported that all three tested medicaments showed predictable outcomes. However, neocone emerged as a superior dressing in terms of faster and sustained pain relief, a smaller number of dental visits for dressing change, and better wound healing. On the other hand, ZOE was found to be the most cost‐effective and easily available medication for dressing.^3^ Kaya et al demonstrated the salicept patch with acemannan as its main ingredient as a viable alternative to alvogyl in the treatment of AO.^35^

The present study demonstrated better pain relief using PRF than ZOE in contrast to previously reported studies.^14^, ^16^ In patients treated with ZOE dressing, the average VAS score observed were 7.4 ± 1.5, 5.1 ± 1.1, 3.4 ± 0.9, and 2.1 ± 0.7, respectively, on the “1st, 3rd, 5th, and 7th” follow‐up days. In patients treated with PRF, the average VAS score observed was 4.1 ± 1.2, 2.6 ± 0.9, 1.7 ± 0.9, and 0.8 ± 0.8, respectively. Both ZOE and PRF were effective for pain control during the follow‐up period. However, the pain intensity measured as a pain score using VAS was, lower in the PRF group than in the ZOE group on all follow‐up days.

The authors attribute the better pain relief in the PRF group to faster wound healing associated with it.^16^, ^27^ The fibrin matrix in PRF promotes angiogenesis and enhances natural immunity, thus reducing inflammatory processes and pain. PRF also provides natural resurfacing of the dry socket wound, which ultimately results in the covering of the exposed nerve endings, thus providing a soothing effect.^31^ Chakravarthi suggested that the kinins released from the dry socket may be antagonized by the various growth factors present in PRF, thereby causing pain relief.^26^

### 5.1 Limitations

Wound healing could not be assessed in this study because of practical difficulties and time constraints. Although a 2‐week follow‐up could have been ideal to assess the pain scores among treatment groups, the follow‐up period was limited to 7 days, considering the risk of patients being lost to follow‐up. Since pain is assessed subjectively using VAS, there is always a possibility of differences in pain perception among individuals based on their pain threshold.

ZOE dressing offers a simple, non‐invasive, conventional, cost‐effective, and handy method, in contrast to PRF, which is minimally invasive, requires special armamentarium, and is more time consuming, along with additional expenses. ZOE dressings can elicit allergic and foreign body reactions, whereas PRF is an autologous biomaterial with less chance of antigenicity and is more patient compliant. ZOE dressings are temporary and need to be changed at frequent intervals for pain relief as the dressings may dislodge or its effect wane over a period. On the other hand, PRF is a permanent autologous substitute secured inside the clot‐less socket with sutures, with minimal chance of dislodging. ZOE dressings leave an unhealed empty socket on the seventh day as bone fill and healing are delayed, while PRF acts as a scaffold for faster healing. However, there are inherent risks such as mismatches and viral and bacterial contamination during the handling of blood products. From the author's experience, PRF is cost‐effective and imparts better healing and pain relief in AO patients.

## 6 CONCLUSION

The maximum number of AO patients were in the age group of 30‐39 years. The incidence of AO was more common in females among the treatment groups. PRF provides better pain reduction than ZOE when used in the management of AO. In patients treated with ZOE dressing, the average VAS score observed were 7.4 ± 1.5, 5.1 ± 1.1, 3.4 ± 0.9, and 2.1 ± 0.7, respectively on the “1st, 3rd, 5th, and 7th” follow up days. In patients treated with PRF, the average VAS score observed was 4.1 ± 1.2, 2.6 ± 0.9, 1.7 ± 0.9, and 0.8 ± 0.8, respectively. PRF in comparison with ZOE is cost‐effective, minimally invasive, carries a low risk of antigenicity, is more patient compliant, and imparts better healing and pain relief. ZOE dressing offers a simple, non‐invasive, conventional, cost‐effective, and handy method.

## CONFLICT OF INTEREST

The authors have no conflicts of interest to declare.

## AUTHOR CONTRIBUTIONS

Conceptualization: Satheesh Reeshma

Data curation: Satheesh Reeshma

Formal Analysis: Satheesh Reeshma, Chacko Pearl Dain

Funding: Satheesh Reeshma

Methodology: Satheesh Reeshma

Resources: Satheesh Reeshma

Software: Chacko Pearl Dain

Supervision: Chacko Pearl Dain

Validation: Chacko Pearl Dain

Visualization: Chacko Pearl Dain

Writing‐original draft: Satheesh Reeshma, Chacko Pearl Dain

Writing‐review and editing: Satheesh Reeshma, Chacko Pearl Dain

S Reeshma and CP Dain have reviewed, discussed, and agreed to our individual contributions in the study.

S Reeshma and CP Dain have full access to all of the data in the study and takes complete responsibility for the integrity of the data and the accuracy of the data analysis.

## TRANSPARENCY STATEMENT

Dr Satheesh Reeshma affirms that this manuscript is an honest, accurate, and transparent account of the study being reported; that no important aspects of the study have been omitted; and that any discrepancies from the study as planned (and, if relevant, registered) have been explained.

## Data Availability

The authors confirm that the data supporting the findings of this study are available within the article [and/or] its supplementary materials.
